# Victory

**Published:** 1892-07-02

**Authors:** 


### PASSING TOPICS.

### VICTORY.

If ever there were a time when the managers of our voluntary hospitals might be expected to regard their critics with complacency, and to allow something to their irritation, it is surely when the enemy's fire is silenced all along the line of his attack, and his discomfiture seems complete.

The enemy has been legion. Everybody with a fad, a grievance, a disappointment, or a sense of dissatisfaction incapable of any sort of plain definition, has helped to swell his ranks. This motley host, recruited day by day from that large body of citizens who would not willingly err, but whose inclinations exercise a masterful albeit unacknowledged influence with their consciences, and who in these days of multifarious appeals, have learned to assimilate gratefully any pretext for refusal, has for some time past stood between our hospitals and their supplies, threatening them with famine, invoking judgment on their imaginary sins, and exulting in their anticipated overthrow.

Not for the first time in the course of history the vaticinations of the over-confident have proved misleading. A great battle has been fought and victory gained, upon an empty stomach. The starving soldiers of the sacred cause of the sick—the cause of "Suffering London"—have prevailed, and although it is too much to assume that the host of adversaries will vanish into thin air, or even put aside their weapons, we may believe that for the time being organised efforts at destruction and spoliation are at an end.

We wish we could feel certain that the difficulties of our medical institutions would cease with their victory, and that they might look forward to a period of peace and plenty. But there is something more to be dreaded than open hostility or even the insidious methods of the conspirator, and that is indifference. Many a lover hopes better things of the hatred of his mistress than of her disdain, and unhappily, whatever theorists may affect to believe, a manifestation, no matter how absolute and complete of the value of work performed, is not sufficient to ensure for it the essentials of existence.

Hospitals want their worth and their needs brought home not only to the reason of the community, but to the hearts of the individuals who compose it. It is no more possible to raise ample funds for supporting the hospitals as they are, and for the necessary development of their work merely by an appeal to duty, than it is to obtain grapes from thorns or figs from thistles. Once more we must insist that for one man who gives because he has persuaded himself, or has been persuaded, that he ought, a hundred men and women will wait until compassion says they must. The events of the past two weeks, and the manifold testimonies to the worth of our voluntary hospitals, contribute a veritable triumph for their managers, but victory may yet prove barren. The ground has been won, but skill and and industry are needed as much as ever to raise the harvest. AGRICOLA.

